# Corneal Confocal Microscopy as a Non-Invasive Marker of Small Fiber Neuropathy and Systemic Complications in Type 2 Diabetes: A Cross-Sectional Study

**DOI:** 10.3390/biom16040483

**Published:** 2026-03-24

**Authors:** Savelia Yordanova, Diana Nikolova, Lachezar Traykov, Antoaneta Gateva, Zdravko Kamenov

**Affiliations:** 1 Department of Internal Medicine, Faculty of Medicine, Medical University-Sofia, 1431 Sofia, Bulgaria; nikolowa.diana@abv.bg (D.N.); tony_gateva@yahoo.com (A.G.); zkamenov@hotmail.com (Z.K.); 2 Department of Neurology, Faculty of Medicine, Medical University-Sofia, 1431 Sofia, Bulgaria; traykov_l@yahoo.fr

**Keywords:** diabetic polyneuropathy, corneal confocal microscopy, small fiber neuropathy

## Abstract

Small fiber neuropathy (SFN) is an early and common manifestation of diabetic polyneuropathy in type 2 diabetes mellitus (T2DM), often presenting with pain, dysesthesia, and autonomic dysfunction. Conventional diagnostic methods primarily assess large nerve fibers and may miss early small fiber damage, while skin biopsy, though considered the reference standard, is invasive. Corneal confocal microscopy (CCM) offers a rapid, noninvasive alternative for visualizing and quantifying small nerve fiber pathology in vivo. This was a monocentric observational study including 80 adults with T2DM (18–75 years), conducted at Alexandrovska Hospital, Sofia. Peripheral neuropathy was evaluated using a modified Neuropathy Disability Score and CCM-derived corneal nerve fiber density (CNFD), length (CNFL), and branching density (CNBD). Autonomic and sudomotor function were assessed by cardiovascular reflex tests and Sudoscan. Additional measures included vibration perception threshold, carotid intima–media thickness, body composition analysis, and laboratory parameters. Autonomic neuropathy was present in 66.7% and peripheral neuropathy in 57.5% of participants. Affected patients were older and had higher BMI and longer diabetes duration; peripheral neuropathy was additionally associated with higher HbA1c. Corneal nerve parameters negatively correlated with diabetes duration, HbA1c, intima–media thickness, and vibration threshold. Patients with diabetic retinopathy showed significantly reduced CNFD and CNFL. ROC analysis demonstrated significant discriminative ability of the HRV index for identifying peripheral neuropathy and of CNFD for detecting sudomotor dysfunction. These findings support CCM as a valuable, noninvasive marker of small fiber damage, closely linked to metabolic control, vascular impairment, and both sensory and autonomic dysfunction in T2DM.

## 1. Introduction

As the global prevalence of diabetes escalates, the burden of chronic complications, particularly diabetic peripheral neuropathy (DPN), is expected to rise markedly. Current management strategies emphasize early detection and prevention of diabetic foot complications. Notably, up to half of patients with DPN experience neuropathic pain, significantly impairing quality of life [1].

Diabetic neuropathy (DN) represents a heterogeneous group of neuropathic disorders and is the most prevalent microvascular complication of T2DM, affecting approximately 50% of patients after ten years of disease duration and 20% at diagnosis. Despite its high prevalence, DN often remains undetected, as nearly 50% of patients are asymptomatic. Clinically, DN encompasses a broad spectrum of manifestations, ranging from peripheral neuropathy to autonomic dysfunction involving the cardiovascular, gastrointestinal, genitourinary, sudomotor, and pupillary systems [2].

Patients with DPN may suffer from neuropathic pain, sensory loss, impaired balance, recurrent falls, foot ulcers, and amputations. Cardiovascular autonomic neuropathy (CAN) represents one of the most severe forms of DN, as it can exacerbate cardiovascular disease and contribute to heart failure and sudden cardiac death. The most significant risk factors include longer diabetes duration, poor glycemic control, and other cardiovascular risk factors such as obesity, hypertension, smoking, and dyslipidemia [1,3]. Detecting autonomic dysfunction in clinical practice could be performed with the HRV (heart variability) index. This geometric measure of heart rate variability provides a robust assessment of overall autonomic function. Analyzing the distribution of NN intervals captures the long-term fluctuations in heart rate while being less affected by noise or missing beats. These measures offer a reliable global indicator of cardiac autonomic health [4].

A simple, generally agreed upon definition of the diabetic neuropathies is “the presence of symptoms and/or signs of peripheral nerve dysfunction in people with diabetes after the exclusion of other causes”. It is also accepted that neuropathy cannot be diagnosed without a careful clinical examination, as the absence of symptoms can never be equated with the absence of neuropathy: asymptomatic neuropathy is common as well. An early clinical classification was published by Boulton and Ward in 1986. The most recent classification that is generally accepted comes from the ADA 2017 Position Statement on Diabetic Neuropathies [5].

Depending on the type of the predominantly involved nerve fibers, diabetic neuropathy is differentiated into small, large and mixed types. Small fiber neuropathy (SFN) is a distinct subtype of peripheral neuropathy characterized by damage to thinly myelinated Aδ fibers and unmyelinated C fibers. These fibers account for approximately 79.6% to 91.4% of peripheral nerve fibers and are responsible for critical functions such as thermal and pain sensation, sudomotor activity, and regulation of tissue blood flow. Dysfunction in these pathways contributes directly to the development of diabetic foot complications, including ulceration. In addition, small fibers play an essential role in autonomic regulation, and their impairment is frequently described as autonomic neuropathy. SFN represents a special problem, because of its high prevalence, earlier development, painful nature, and “silent” conventional electrophysiological tests (normal nerve conduction studies and electromyography), which are only appropriate for large fibers [6,7].

In 2010, the Diabetic Neuropathy Study Group of the European Association for the Study of Diabetes (NEURODIAB) proposed diagnostic criteria for SFN in diabetes based on a grading as: (i) possible, if symptoms or clinical signs of small fiber damage or both; (ii) probable, if clinical signs of small fiber damage and normal sural NCS; and (iii) definite, if clinical signs of small fiber damage, normal sural NCS, and abnormal QST thresholds at the foot, reduced IENFD at the ankle or both. The NEURODIAB criteria do not require specification of the clinical signs of small fiber damage. Despite advances in detecting SFN across the spectrum of dysglycemia, including prediabetes, type 1 diabetes (T1DM), and type 2 diabetes (T2DM), these criteria have not been revised. They remain centered on length-dependent symptoms, intraepidermal nerve fiber density (IENFD) from skin biopsy, and quantitative sensory testing (QST) [8].

Small fiber integrity can be assessed from two complementary perspectives. Functional evaluation measures the physiological performance of these fibers and includes methods such as quantitative sensory testing, sudomotor assessments, laser Doppler imaging flare and evoked potentials. These tests provide insight into the ongoing functional status of the fibers, but their specificity may be influenced by factors such as age, medications, comorbidities, and patient cooperation. In contrast, structural evaluation offers direct visualization of small fibers, allowing for quantification of fiber density, length, and branching. Techniques such as skin biopsy with intraepidermal nerve fiber density assessment or corneal confocal microscopy (CCM) provide objective evidence of fiber loss independent of systemic or behavioral influences. By incorporating both functional and structural assessments, clinicians and researchers can gain a more comprehensive understanding of small fiber health, improving the accuracy of SFN diagnosis, monitoring disease progression, and evaluating response to therapeutic interventions [9,10,11].

Relatively few studies have comprehensively integrated structural corneal confocal microscopy (CCM) assessments with functional autonomic testing and systemic complication profiling in patients with type 2 diabetes mellitus (T2DM). Most existing research focuses on these domains separately, limiting the understanding of how structural nerve alterations correlate with functional impairment and broader systemic involvement.

The cornea is the most sensitive structure in the human body. It is a transparent and avascular component of the eye that is densely innervated by sensory fibers from the ophthalmic branch of the trigeminal nerve, which mediate reflexes, tear production, and blinking while maintaining ocular surface integrity through neurotrophic support (via neurotrophins and neuromediators). Corneal nerves enter via the limbus, forming a circumferential plexus from which fibers penetrate the stroma, run parallel to collagen lamellae, and form the mid-stromal plexus. Fibers then ascend through Bowman’s membrane to create the subbasal nerve plexus (SBNP), displaying a centripetal whorl-like pattern beneath the epithelium. Human corneal innervation comprises small myelinated A-delta and unmyelinated C fibers, predominantly polymodal nociceptors (~70%), with mechanoreceptors (~20%) and cold receptors (~10%). Sensory fibers convey thermal, mechanical, and chemical stimuli, driving the blink reflex and reflex tearing via parasympathetic pathways. Sympathetic autonomic fibers accompany sensory nerves in the limbal plexus, regulating tear film secretion and vasomotor tone, highlighting the cornea’s integrated role in sensory and autonomic ocular function [12,13].

Corneal confocal microscopy (CCM) was first proposed by Prof. R. Malik et al. in 2003 as a novel, non-invasive technique to visualize small corneal nerve fibers in patients with diabetic neuropathy [14]. Since its introduction, CCM has continuously been refined and developed, establishing it as a reliable and sensitive tool for assessing small fiber neuropathy in diabetes and various other neurological disorders [15,16].

Integrating CCM, functional autonomic testing and systemic complication assessment could yield a better understanding of diabetic neuropathy, enhance early detection, and facilitate risk stratification for complications. This multidimensional evaluation strategy represents an important step toward precision medicine in diabetic neuropathy research.

The aim of this study was to investigate structural alterations to small nerve fibers in patients with type 2 diabetes, using CCM, and their relationship with functional measures of peripheral and autonomic nerve function, as well as major diabetic complications.

## 2. Materials and Methods

### 2.1. Study Design

This study was designed as a monocentric, cross-sectional, observational investigation conducted at the Endocrinology and Metabolic Disorders Clinic of Alexandrovska Hospital. A total of 80 participants diagnosed with type 2 diabetes mellitus (T2DM) were consecutively recruited. Eligible participants were between 18 and 75 years of age, had a confirmed diagnosis of T2DM for at least one year, and were capable of providing informed consent. Individuals were excluded if they had coexisting ocular conditions (such as keratoconus or glaucoma), a history of eye surgery, neurological diseases unrelated to diabetes, chronic alcohol consumption, or other disorders known to impair peripheral nerve function. The study protocol received approval from the Ethics Committee of Medical University of Sofia (Approval No. 4439/5 July 2022). All procedures adhered to institutional ethical requirements and the principles outlined in the Declaration of Helsinki. Written informed consent was obtained from all participants prior to inclusion.

### 2.2. Diagnosis of Neuropathy

#### 2.2.1. Clinical Neuropathy Assessment (Modified NDS)

Peripheral sensory function was assessed using a version of the neuropathy disability score (NDS) modified for lower limbs. The evaluation included pinprick sensation (using a disposable neurotip), thermal sensation (using warm/cold stimulus), vibration perception (using a 128 Hz-tuning fork, applied to the hallux) and ankle reflex testing with a reflex hammer. Each sensory modality (vibration, temperature, and pinprick) was scored as 0 = present (normal) or 1 = absent/reduced for each foot (maximum 2 points per modality). Ankle reflexes were graded as 0 = normal, 1 = present with reinforcement, or 2 = absent, for each side. The total NDS ranged from 0 to 10 points, with higher scores indicating greater neurological impairment. In accordance with previously published thresholds, an NDS > 5 was considered indicative of clinically significant peripheral neuropathy. Higher scores indicated greater neuropathic impairment [17].

#### 2.2.2. Peripheral Neuropathy Assessment by Corneal Confocal Microscopy (CCM)

Small fiber neuropathy was further evaluated using corneal confocal microscopy (Heidelberg Retinal Tomograph III with Rostock Cornea Module, Heidelberg Engineering, Heidelberg, Germany). Imaging was performed according to standardized acquisition protocols. Each patient had a total of 6 (3 of each eye) high-quality, well-focused images of the central corneal subbasal nerve plexus analyzed. Quantitive parameters included: corneal nerve fiber density (CNFD)—fibers per mm^2^; corneal nerve branch density (CNBD)—branches per mm^2^; corneal nerve fiber length (CNFL)—total fiber length per mm^2^. The investigator was not blinded to the clinical data, but images were analyzed using automated software (ACCMetrics, version 2.0), which minimized observer-dependent variability. Diagnostic thresholds for corneal nerve parameters were defined according to the normative values established by Malik et al [18].

#### 2.2.3. Autonomic Neuropathy Assessment

Autonomic function was measured using a Cardiosys Extra system under standardized resting conditions to minimize variability. The scoring was based on standardized Ewing cardiovascular reflex tests. Following the eighth international symposium on diabetic neuropathy in 2010, criteria for diagnosis and staging CAN are defined in the CAN Subcommittee of the Toronto Consensus Panel statement. Accordingly, only one abnormal CART result is sufficient to diagnose possible or early CAN; among the seven autonomic function analyses (5 CART, time-domain and frequency-domain HRV test), two or three abnormal tests (points) indicate definite or confirmed CAN; and severe CAN can be indicated by concurrent orthostatic hypotension. In our study, a total score of ≥3 points was classified as a positive finding for autonomic neuropathy [19].

Participants fulfilling criteria for both peripheral and autonomic neuropathy were categorized as having combined neuropathy.

#### 2.2.4. Sudomotor Function

Sudomotor function was assessed using (Sudoscan^®^, Impeto Medical, Saint-Denis, France). Electrochemical skin conductance (ESC) values were obtained from hands and feet and expressed in microsiemens (µS), providing an indirect measure of sympathetic sudomotor activity and small fiber function.

### 2.3. Laboratory Investigations

#### Blood Sampling

Fasting venous blood samples were obtained and collected in EDTA-containing tubes. Plasma was isolated by centrifugation and stored at −80 °C until analysis.

### 2.4. Statistical Analysis

Statistical analyses were performed using SPSS 23 software. Data are presented as mean ± standard deviation (SD) for normally distributed variables. Distribution normality was assessed using the Shapiro–Wilk or Kruskal–Wallis tests. Comparisons between two groups were conducted using independent samples *t*-tests, while one-way ANOVA was applied for comparisons involving more than two groups, followed by Bonferroni post hoc correction when appropriate. Non-parametric alternatives (Mann–Whitney U test or Kruskal–Wallis test) were used when normality assumptions were not met. Correlations between continuous variables were evaluated using Pearson’s correlation coefficient. A *p*-value < 0.05 was considered statistically significant. Potential confounding factors—including body mass index (BMI), renal function (eGFR), smoking status, and antidiabetic therapies (e.g., metformin, insulin, GLP-1 receptor agonists, SGLT-2 inhibitors)—were recorded and examined. Due to sample size constraints, not all variables could be simultaneously included in multivariable models, which may introduce residual confounding.

## 3. Results

### 3.1. Participants

The average age of the participants was 59.5 ± 7.9 years. The mean duration of diabetes was 8.8 ± 5.7 years.

### 3.2. Treatment

**Figure 1:**
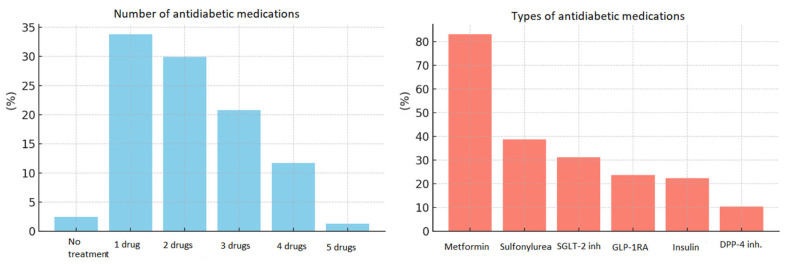
Treatment characteristics of the patients. Blue bars represent the percentage of patients according to the number of antidiabetic medications used (from no treatment to five drugs). Red bars represent the percentage of patients using each type of antidiabetic medication.

Most participants were receiving pharmacological treatment for diabetes, with only 2.5% not on any therapy. Monotherapy was reported in 33.8% of cases, while 29.9% were treated with two agents, 20.8% with three, and 11.7% with four medications; one participant was on a regimen consisting of five drugs. Metformin was the most commonly found medication (83.1%), followed by sulfonylureas (38.7%), SGLT-2 inhibitors (31.2%), GLP-1 receptor agonists (23.7%), insulin (22.4%), and DPP-4 inhibitors (10.5%). Treatment characteristics of patients are shown in Figure 1.

### 3.3. Diabetes Complications

The prevalence of diabetic complications in the overall cohort was as follows: diabetic peripheral neuropathy (DPN)—57.5% (assessed via corneal confocal microscopy), autonomic neuropathy (DAN)—66.7%, nephropathy—27.3%, retinopathy—14.3%, coronary artery disease (CAD)—18.4%, history of myocardial infarction—11.8%, stroke—5.3%, and peripheral arterial disease (PAD)—5.3%. The presence of macrovascular complications such as coronary artery disease and myocardial infarction further reflects the systemic vascular burden in this cohort, supporting the concept that small fiber neuropathy and vascular dysfunction share common metabolic and endothelial mechanisms.

Characteristics of patients with and without DAN are presented in Table 1. Patients with diabetic autonomic neuropathy (DAN) were slightly older and had a higher BMI and waist-to-hip ratio compared to those without DAN. The duration of diabetes was longer in patients with DAN as well. Overall, there was a trend toward older age and longer diabetes duration in patients with DAN compared to those without.

**Figure 2:**
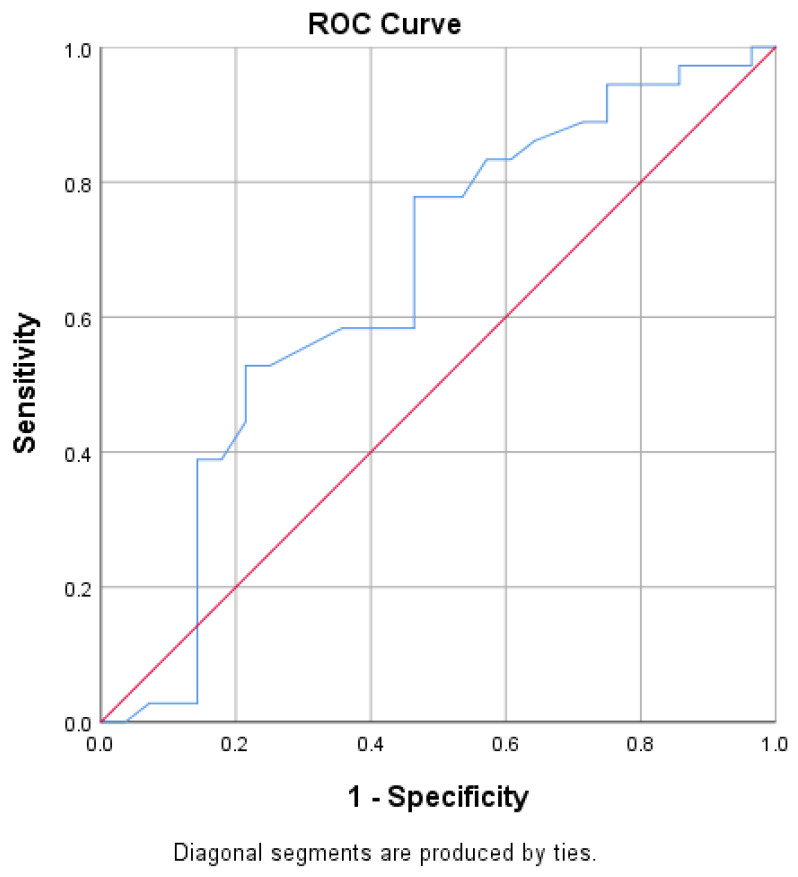
Predictive value of HRVi for the presence of DPN (assessed via CCM). The blue curve represents the receiver operating characteristic (ROC) curve of HRVi for detecting DPN, illustrating the relationship between sensitivity and 1-specificity across different thresholds. The red diagonal line indicates the performance of a random classifier. The position of the ROC curve above the diagonal suggests that HRVi has discriminatory ability in identifying DPN.

The ROC analysis demonstrated that HRVi (heart variability index) had statistically significant discriminative ability for identifying peripheral neuropathy confirmed by corneal confocal microscopy (AUC = 0.652, 95% CI: 0.511–0.793, *p* = 0.038) (Figure 2 and Table 2).

Patients with sudomotor dysfunction (defined as ANR > 50) had significantly higher fat percentage (40.47 ± 6.78 vs. 32.44 ± 7.55, *p* < 0.0001), fat mass (44.09 ± 12.44 vs. 29.92 ± 10.83, *p* < 0.0001), and body weight (110.77 ± 18.83 vs. 92.15 ± 15.79 kg, *p* = 0.0001) compared with those with ANR ≤ 50. Total body water percentage was lower in the ANR > 50 group (41.37 ± 5.30 vs. 46.61 ± 6.04, *p* = 0.0003), while visceral fat rating was significantly higher (16.63 ± 5.70 vs. 11.77 ± 4.47, *p* = 0.0004). Corneal nerve fiber density (CNFD) was lower in patients with ANR > 50 (15.84 ± 5.94 vs. 19.02 ± 5.55, *p* = 0.0343).

**Figure 3:**
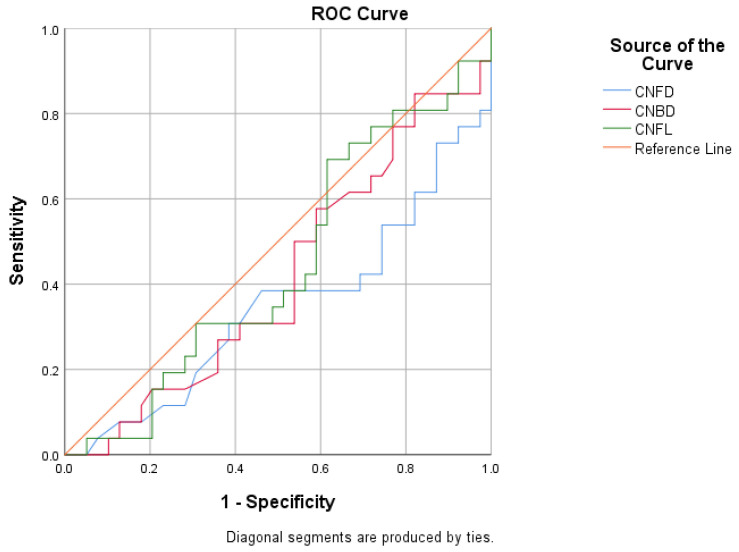
Negative association of CNFD with the presence of sudomotor dysfunction.

ROC curve analysis demonstrated a statistically significant inverse association between CNFD and sudomotor dysfunction (AUC = 0.347, 95% CI: 0.206–0.487, *p* = 0.037). The AUC below 0.5 indicates that lower CNFD values are associated with a higher probability of sudomotor dysfunction, reflecting an inverse relationship between corneal nerve loss and autonomic impairment (Figure 3 and Table 3).

Characteristics of patients with DPN are summarized in Table 4. Patients with DPN were older and had a higher BMI compared to those without DPN. Waist-to-hip ratio was similar between groups, while waist-to-stature ratio was slightly higher in patients with DPN. Diabetes duration was significantly longer in the DPN group (10.4 ± 6.0 vs. 6.8 ± 4.7 years, *p* < 0.05). HbA1c levels were also higher in patients with DPN (7.9 ± 1.4 vs. 7.3 ± 1.1%, *p* < 0.05).

A significant negative correlation was observed between diabetes duration and CNFD (r = −0.306, *p* = 0.011) as well as CNFL (r = −0.308, *p* = 0.010), and between HbA1c and CNFD (r = −0.311, *p* = 0.01).

**Figure 4:**
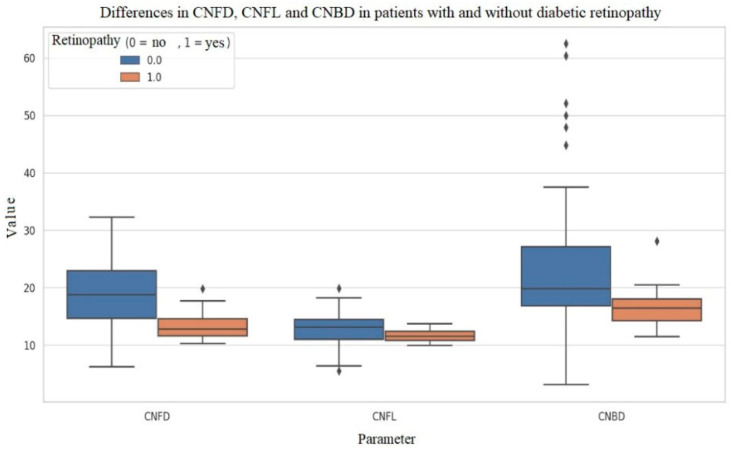
Differences in CNFD, CNFL and CNBD in patients with and without diabetic retinopathy. The horizontal line within each box represents the median, while the boxes and whiskers indicate the interquartile range and 95% confidence intervals, respectively. Individual rhombic symbols (⧫) denote statistical outliers falling beyond 1.5 times the interquartile range.

Patients with diabetic retinopathy had significantly lower CNFD (13.5 ± 2.9 no./mm^2^ vs. 18.9 ± 5.8, *p* < 0.001) and CNFL (11.6 ± 1.2 mm/mm^2^ vs. 12.8 ± 2.7, *p* = 0.026) compared to those without retinopathy (Figure 4). Such differences were not observed for diabetic nephropathy or DAN.

**Figure 5:**
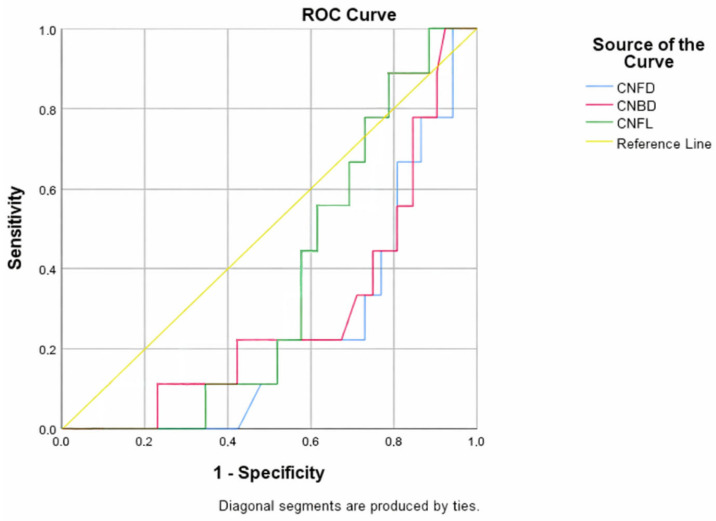
Negative association of CCM parameters with the presence of diabetic retinopathy.

ROC analysis demonstrated a statistically significant inverse association between CCM parameters and diabetic retinopathy (Figure 5 and Table 5). CNFD and CNBD showed weak but significant discriminatory ability (CNFD AUC = 0.240, *p* = 0.013; CNBD AUC = 0.287, *p* = 0.043), indicating that lower corneal nerve values were associated with a higher likelihood of diabetic retinopathy.

**Figure 6:**
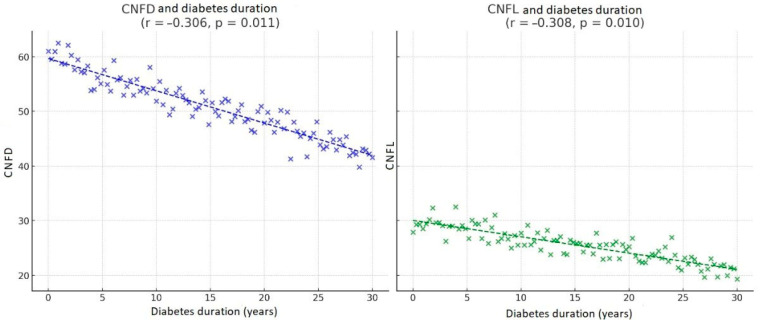
Correlations between CNFD, CNFL and diabetes duration.

A significant negative correlation was observed between CNFD and CNFL and diabetes duration (r= −0.306, *p* = 0.011 and r= −0.308, *p* = 0.010 respectively) (Figure 6).

**Figure 7:**
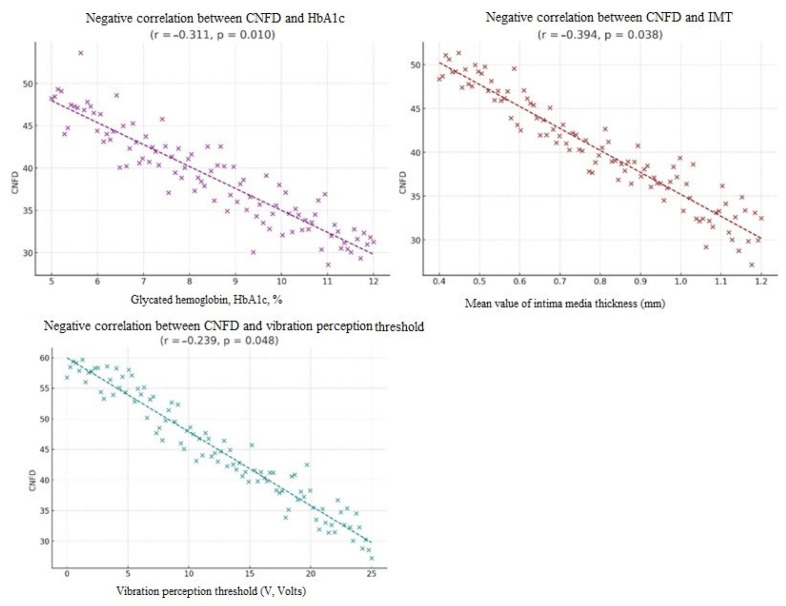
Correlations between CNFD and HbA1c, IMT and VPT.

We also found a negative correlation between CNFD and HbA1c (r = −0.311, *p* = 0.010), carotid intima–media thickness (r = −0.394, *p* = 0.038), and vibration perception threshold (r = −0.239, *p* = 0.048) (Figure 7).

## 4. Discussion

Diabetic neuropathy represents one of the most prevalent and debilitating complications of type 2 diabetes mellitus (T2DM), affecting both somatic and autonomic fibers. Evidence suggests that small fiber neuropathy (SFN) develops early in the course of diabetes and may precede clinically detectable large fiber or autonomic dysfunction [20]. In this context, corneal confocal microscopy (CCM) has emerged as a sensitive, noninvasive imaging technique that enables direct visualization and quantification of small unmyelinated C-fibers and thinly myelinated Aδ-fibers within the corneal subbasal nerve plexus. Our findings reinforce the diagnostic and prognostic potential of CCM, demonstrating significant associations between structural small fiber changes and multiple diabetic complications, including peripheral neuropathy, autonomic dysfunction, and microvascular disease. The study, however, has some limitations. The modest sample size limits statistical power, and not all potential confounders (e.g., BMI, renal function, smoking status, and glucose-lowering medications) could be included simultaneously in multivariable models. Therefore, the observed associations between CCM parameters and diabetic complications should be interpreted cautiously. The monocentric recruitment from a specialized endocrinology clinic may introduce selection bias and restrict the generalizability of the results to the wider T2D population. Larger, longitudinal studies with full adjustment for metabolic and clinical factors are needed to confirm the predictive value of CCM for neuropathy progression and systemic complications.

The associations observed in our cohort between corneal nerve morphology, glycemic control, and disease duration align closely with established longitudinal and meta-analytical data. Specifically, our findings regarding reduced CNFL as a hallmark of neuropathy are consistent with Perkins et al. (2021) [21], who demonstrated that baseline reductions in CNFL significantly precede the clinical onset of DPN. This supports that corneal confocal microscopy (CCM) could serve not only as a diagnostic tool but also as a sensitive biomarker associated with early nerve fiber loss, potentially identifying patients at higher risk of neuropathy [21]. Moreover, Ponirakis and colleagues found that elevated HbA1c is linked to sustained nerve fiber loss, which supports our results regarding HbA1c and CNFD [22]. A recent meta-analysis by Vidyasagar et al. (2025) also confirmed marked reductions in CNFL, CNFD, and CNBD in patients with DPN, especially in painful neuropathy forms [23]. Additionally, a community-based screening study reported that longer diabetes duration and elevated HbA1c correlated with greater abnormalities in CCM-derived nerve parameters, suggesting a cumulative metabolic impact on nerve integrity [24].

Patients with sudomotor dysfunction (ANR > 50) had significantly lower CNFD, indicating a link between autonomic dysfunction and corneal nerve loss. They also exhibited higher fat mass, body weight, and visceral fat rating, suggesting a potential link between obesity and autonomic small fiber impairment. BMI may influence autonomic and small fiber function, but the relationship is likely complex and influenced by regional and systemic factors, highlighting the need for further investigation [25,26]. In 2015, Tavakoli and colleagues evaluated the utility of corneal confocal microscopy (CCM) as a diagnostic tool for diabetic autonomic neuropathy (DAN). In their study, patients with established DAN—as defined by elevated Composite Autonomic Scoring Scale (CASS) scores—exhibited significantly reduced corneal nerve fiber density, branch density, and length compared to both healthy controls and diabetic patients without DAN. These corneal nerve parameters correlated strongly with clinical measures of autonomic dysfunction, demonstrating CCM’s high sensitivity and specificity for identifying both subclinical and overt DAN [27]. Furthermore, Azmi et al. (2025) demonstrated progressive and significant reductions in deep breathing–heart rate variability (DB-HRV), expiration–inspiration (E:I) ratio, 30:15 ratio, corneal nerve fiber density (CNFD), corneal nerve branch density (CNBD) and corneal nerve fiber length (CNFL) with increasing severity of CAN [28]. Our ROC analysis demonstrated a statistically significant inverse association, where lower CNFD values were associated with the presence of sudomotor dysfunction. This inverse relationship is consistent with the expected direction—lower CNFD corresponding to greater autonomic impairment—suggesting that CCM captures the same small fiber pathology that contributes to both sensory and autonomic manifestations of diabetic neuropathy. Ishibashi et al. demonstrated that reduced corneal nerve fiber density parallels impaired sudomotor function in type 2 diabetes. Their study showed that abnormalities in sweat gland innervation and corneal nerve loss move in the same direction—both reflecting small fiber degeneration. Thus, our inverse association directly supports and extends the published evidence that CCM captures the same small fiber pathology responsible for autonomic impairment [29].

The presented characteristics of patients with and without DAN reflect the systemic metabolic burden associated with autonomic fiber injury. Such findings underscore the clinical importance of identifying early small fiber pathology before irreversible autonomic dysfunction develops. The combined use of CCM and functional autonomic assessments—such as HRV and sudomotor testing—offers a comprehensive, structure–function framework for evaluating diabetic neuropathy, allowing earlier diagnosis and risk stratification. The discriminative performance of HRVi for DPN underlines the link between autonomic dysfunction and small fiber pathology. Reduced heart rate variability is a well-established marker of cardiovascular autonomic neuropathy and is largely mediated by small autonomic fibers. Its significant association with DPN in this study indicates a shared pathophysiological substrate between somatic and autonomic small fiber loss. Taken together, these findings position CCM as a key structural correlate of both peripheral and autonomic small fiber dysfunction.

Corneal nerve loss was not limited to neuropathic manifestations but also correlated with other systemic and microvascular complications. Patients with diabetic retinopathy exhibited significantly lower CNFD and CNFL compared with patients without retinopathy. These findings highlight the systemic nature of microvascular complications in diabetes and emphasize the value of corneal confocal microscopy (CCM) in detecting early subclinical damage. Recent evidence confirms that CCM-derived parameters, including CNFD reduction in corneal nerve and CNFL were also reduced and showed a significant negative correlation with the presence of fundus lesions in the retina, suggesting their utility as non-invasive biomarkers of retinal microvascular disease [30]. Our results also show a weak but statistically significant negative association of CNFD and CNBD with the presence of diabetic retinopathy. Earlier studies reported as well that corneal nerve loss assessed by CCM correlates with the severity of diabetic microvascular complications, including both neuropathy and retinopathy, supporting its role as a surrogate marker of systemic microvascular involvement in diabetes [31].

The correlation between CNFD and carotid intima–media thickness further supports a systemic vascular–neural link, indicating that corneal small fiber loss may reflect widespread endothelial dysfunction and vascular injury. In addition, CNFD correlated negatively with vibration perception threshold, demonstrating that structural corneal changes align with functional sensory deficits. This convergence of structural, functional, and vascular indicators strengthens the role of CCM as a surrogate marker for multi-system involvement in diabetes. Our findings showing that CNFD correlates negatively with carotid intima–media thickness and vibration perception threshold align closely with previous work demonstrating that corneal nerve loss is linked to both vascular injury and sensory dysfunction. The studies by Ponirakis et al. similarly reported that reduced CCM parameters were associated with increased IMT and impaired vibrotactile sensation, reinforcing the concept that small fiber degeneration reflects parallel vascular and large fiber abnormalities [32,33]. Together, these external findings strengthen the interpretation of our results and support CCM as a sensitive, multisystem biomarker in diabetes.

## 5. Conclusions

In summary, our findings demonstrate that corneal nerve loss assessed by CCM is strongly linked to both peripheral and autonomic small fiber dysfunction, as well as to systemic metabolic and microvascular abnormalities in type 2 diabetes. Reduced CNFD and CNFL correlate with longer diabetes duration, poor glycemic control, and vascular damage, while also predicting autonomic and sensory deficits. Importantly, the use of CCM offers a noninvasive, rapid, and reproducible means to visualize small fiber pathology—something traditional autonomic or electrophysiological tests cannot achieve at a structural level. When combined with functional autonomic testing (e.g., HRV analysis, sudomotor function), CCM could significantly improve early detection and phenotypic characterization of SFN before large fiber or overt autonomic deficits emerge. This approach could allow clinicians to capture both the structural (nerve loss) and functional (autonomic dysregulation) dimensions of neuropathy. Our findings reinforce the value of CCM as a noninvasive tool for detecting early-stage nerve pathology, providing a structural dimension to the assessment of systemic diabetic complications.

## Figures and Tables

**Table 1:** Characteristics of patients with and without DAN.

Characteristics of Patients With and Without DAN		
	Without DAN	With DAN
Age (y)	58.7 ± 9.1	59.4 ± 8.0
BMI (kg/m^2^)	35.2 ± 5.0	35.9 ± 6.4
WHR	0.94 ± 0.06	1.01 ± 0.14
WSR	0.63 ± 0.16	0.53 ± 0.30
Diabetes duration (y)	8.1 ± 3.9	9.9 ± 6.5

**Table 2:** Predictive value of HRVi for the presence of DPN (assessed via CCM). ^a^—Standard error: The estimated standard deviation of the AUC, reflecting the variability or precision of the AUC estimate; ^b^—Asymptotic significance: The *p*-value testing whether the AUC is significantly different from 0.5 (i.e., no discriminative ability). A value below 0.05 indicates statistically significant discrimination.

Area Under the Curve					
Test Result Variable(s)	Area	Std. Error ^a^	Asymptotic Sig. ^b^	Asymptotic 95% Confidence Interval	
				Lower Bound	Upper Bound
HRVi	0.652	0.072	0.038	0.511	0.793

**Table 3:** Negative association of CNFD with the presence of sudomotor dysfunction. ^a^—Standard error: The estimated standard deviation of the AUC, reflecting the variability or precision of the AUC estimate; ^b^—Asymptotic significance: The *p*-value testing whether the AUC is significantly different from 0.5 (i.e., no discriminative ability). A value below 0.05 indicates statistically significant discrimination.

Area Under the Curve					
Test Result Variable(s)	Area	Std. Error ^a^	Asymptotic Sig. ^b^	Asymptotic 95% Confidence Interval	
				Lower Bound	Upper Bound
CNFD	0.347	0.072	0.037	0.206	0.487
CNBD	0.417	0.072	0.258	0.276	0.557
CNFL	0.443	0.073	0.437	0.301	0.585

**Table 4:** Characteristics of patients with and without DPN. *—statistically significant difference compared to the “Without DPN” group (*p* < 0.05).

Characteristics of Patients With and Without DPN		
	Without DPN	With DPN
Age (y)	58.6 ± 8.17	60.7 ± 7.39
BMI (kg/m^2^)	34.8 ± 5.8	36.9 ± 6.3
WHR	0.99 ± 0.15	0.98 ± 00.8
WSR	0.55 ± 0.28	0.59 ± 0.23
Diabetes duration (y)	6.8 ± 4.7	10.4 ± 6.0 *
HbA1c (%)	7.3 ± 1.1	7.9 ± 1.4 *

**Table 5:** Negative association of CCM parameters with the presence of diabetic retinopathy. ^a^—Standard error: The estimated standard deviation of the AUC, reflecting the variability or precision of the AUC estimate; ^b^—Asymptotic significance: The *p*-value testing whether the AUC is significantly different from 0.5 (i.e., no discriminative ability). A value below 0.05 indicates statistically significant discrimination.

Area Under the Curve					
Test Result Variable(s)	Area	Std. Error ^a^	Asymptotic Sig. ^b^	Asymptotic 95% Confidence Interval	
				Lower Bound	Upper Bound
CNFD	0.240	0.070	0.013	0.104	0.377
CNBD	0.287	0.086	0.043	0.120	0.455
CNFL	0.363	0.074	0.193	0.218	0.508

## Data Availability

The original contributions presented in this study are included in the article. Further inquiries can be directed to the corresponding author.
